# Evaluation of the Chewing Pattern through an Electromyographic Device

**DOI:** 10.3390/bios13070749

**Published:** 2023-07-20

**Authors:** Alessia Riente, Alessio Abeltino, Cassandra Serantoni, Giada Bianchetti, Marco De Spirito, Stefano Capezzone, Rosita Esposito, Giuseppe Maulucci

**Affiliations:** 1 Metabolic Intelligence Lab, Department of Neuroscience, Università Cattolica del Sacro Cuore, Largo Francesco Vito, 1, 00168 Rome, Italy; alessia.riente@unicatt.it (A.R.); alessio.abeltino@unicatt.it (A.A.); cassandra.serantoni@unicatt.it (C.S.); giada.bianchetti@unicatt.it (G.B.); marco.despirito@unicatt.it (M.D.S.); 2 Fondazione Policlinico Universitario “A. Gemelli” IRCCS, 00168 Rome, Italy; 3 Gruppo Fastal Blu Sistemi, Via Nomentana 263, 00161 Rome, Italy; stefano@capezzone.it; 4 Digital Innovation Hub Roma, Chirale S.r.l., Via Ignazio Persico 32-46, 00154 Rome, Italy; rosita.esposito@chirale.it

**Keywords:** mastication, chewing profile, EMG device, smoking, chewing features, statistical analysis

## Abstract

Chewing is essential in regulating metabolism and initiating digestion. Various methods have been used to examine chewing, including analyzing chewing sounds and using piezoelectric sensors to detect muscle contractions. However, these methods struggle to distinguish chewing from other movements. Electromyography (EMG) has proven to be an accurate solution, although it requires sensors attached to the skin. Existing EMG devices focus on detecting the act of chewing or classifying foods and do not provide self-awareness of chewing habits. We developed a non-invasive device that evaluates a personalized chewing style by analyzing various aspects, like chewing time, cycle time, work rate, number of chews and work. It was tested in a case study comparing the chewing pattern of smokers and non-smokers, as smoking can alter chewing habits. Previous studies have shown that smokers exhibit reduced chewing speed, but other aspects of chewing were overlooked. The goal of this study is to present the device and provide additional insights into the effects of smoking on chewing patterns by considering multiple chewing features. Statistical analysis revealed significant differences, as non-smokers had more chews and higher work values, indicating more efficient chewing. The device provides valuable insights into personalized chewing profiles and could modify unhealthy chewing habits.

## 1. Introduction

Chewing is a fundamental regulator of the entire metabolic process [1] (cap 22, pp. 716–743). When food is introduced into the oral cavity, the mechanical activity induced by the masticatory muscles and the concomitant salivary secretion (salivary amylase and small quantities of lipase) dissolve the chemical substances present in the food, starting the chemical digestion of some macronutrients. This phase of digestion is important as it triggers an anticipatory response of subsequent sections of the digestive system known as the cephalic phase [1] (cap 22, pp. 716–743), conditioning and regulating the whole digestive process and nutrient absorption.

In the literature, there are several works whose purpose was the analysis of chewing. Various aspects of this activity can be evaluated [2], for example, the force exerted by the masticatory muscles [2,3,4]. Frequently, dental implants with strain gauges are used [4]. However, these devices could alter oral sensation and cannot be used for long-term monitoring. In some solutions, the sounds emitted during the act of chewing are analyzed using acoustic sensors and microphones [5,6,7]. The collected data of these devices suffer from noisy components due to the surrounding environment. Another possible approach consists of measuring the contraction of the masseter and temporal muscles (the main muscles of chewing). For this purpose, piezoelectric sensors [8,9,10] are used. Piezoelectric sensors produce a voltage value when subjected to physical stress [8]. They are, therefore, suitable for detecting muscular activities, such as chewing and even swallowing. A wearable piezoelectric strain sensor in the form of glasses positioned on the temporalis muscle was engineered in [9] to detect chewing features. Similarly, in [10], a method was presented for the automatic quantification of chewing episodes captured by a piezoelectric sensor system. Also, in [8] a device was realized using a necklace with a piezoelectric sensor positioned at the throat level to recognize chewing activity. Although these examples use piezoelectric sensors for chewing analysis, piezoelectric sensors are not able to reliably distinguish movements of masticatory activity from head and neck movements unrelated to this act [11]. Some devices exploit photoplethysmography (PPG) technology [12,13] to detect changes in reflected light levels due to altered venous blood characteristics [14]. The lower jaw moves to open and close the mouth, causing the ear canal to expand as the mandibular condyle slides back and forth. A PPG signal is not completely free of noise; indeed, sudden changes in ambient lighting can produce significant artefacts and cause signal saturation [14].

Among the options mentioned, surface electromyography (EMG) [15,16,17,18] is the most useful and accurate solution for practical purposes. However, standard EMG practice has not been established, and some problems remain [14]. One of the disadvantages of using EMG is the requirement for the direct attachment of sensors to the skin [19]. In [15], the authors exploited EMG technology to analyze the variations in jaw movements when the subject varied the masticatory region, while the authors of [16] presented a 3D glass with bilateral EMG electrodes placed on the temporal muscles. The purpose of this device is to automatically monitor the subject’s diet by recognizing the chewing act and classifying the data relating to the ingested food, obtaining good performance. In [20], an EMG system capable of identifying masticatory events from other activities and their duration was presented. The invention presents an interface with various sections in which different characteristics of the act are analyzed. For example, there is a section capable of determining the maximum value reached by the recorded signal.

The devices described above, which exploit EMG technology, analyze mastication to ascertain the presence of the act or to classify foods based on the masticatory signal. However, no electromyographic device describes chewing performance in its entirety with the aim of defining a precise chewing profile of the user, making him self-aware of his chewing habits to eventually modify them. To obtain more insights into the chewing process, we implemented ‘Chewing’, a device representing a solution to evaluate the chewing style, as it can characterize the masticatory activity in a non-invasive way. ‘Chewing’ analyzes mastication considering different aspects of the process: the chewing time, cycle time, work rate, number of chews and masticatory work. In this way, it is possible to analyze the masticatory process itself, considering different aspects of it, and define personalized chewing profiles. We tested our device by performing a case study in which the chewing profile of smokers and non-smokers was analyzed and identified. Smoking can indeed alter a person’s chewing pattern. The constant exposure to tobacco chemicals leads to damage to oral tissue, lesions of the lips and mucous membranes, loss of teeth and a weakening of the perception of taste and smell [21,22,23], leading to a change in the chewing pattern. In [22], the presence of oral alterations in smokers was verified, and the impact of the alterations on masticatory function was evaluated compared to subjects who have never smoked. However, the variables considered in the study were halitosis, malocclusion, chewing speed and others. It was noted that the chewing speed was significantly reduced in smokers. Similarly, in [24], the authors identified differences in the chewing patterns between smokers and non-smokers considering aspects such as the chewing speed, atypical muscle contractions, and orbicularis and mental contraction muscles during swallowing. Also, in this case, considering the masticatory speed, it has been verified that smokers have a slower pattern. However, in these previous works, only the masticatory speed was evaluated, and other chewing features were neglected. Our aim is, therefore, to add additional insights to this important case study.

## 2. Materials and Methods

### 2.1. Development of a Device to Assess Chewing Behavior

The masticatory behavior is evaluated using the Chewing device, which uses electromyographic technology. Electromyography (EMG) is a technique that allows the measurement of the electrical activity produced by muscles during contraction [25]. Since the masseter and temporal muscles are the most important muscles in mastication, the first one was chosen for monitoring as it is more easily accessible. The signal is picked up using surface electrodes, which are commonly used with this technique. These electrodes are made of conductive materials, such as silver or silver chloride, which are characterized by high electrical conductivity and can adhere to the skin securely.

**Figure 1:**
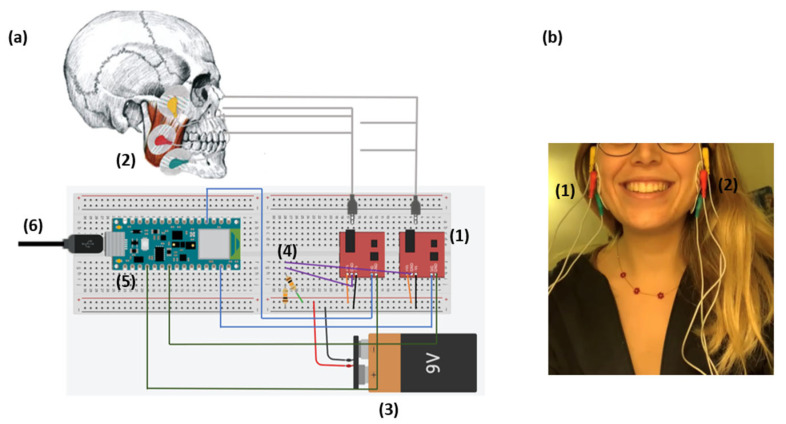
Chewing device. (**a**) Scheme of the device and all the parts that it includes: (5) Arduino nano 33 BLE microprocessor connected to a PC via cable (6), two Arduino muscle v3 modules (1) connected to the microprocessor through a resistive divider (4) and a 9 volt battery (3). The signal is taken through the electrodes (2) connected to the Arduino muscle v3 modules. (**b**) Placement of EMG electrodes on both the masseters of a subject (1–2): the red ones on the central part of the muscles, the green ones at the end of the masseters and the yellow ones on the cheekbones.

This device consists of an Arduino nano BLE 33 microprocessor (Microcontroller: nRF52840, Operating Voltage: 3.3 V, DC Current per I/O Pin: 15 mA, Length: 45 mm, Width: 18 mm, Weight: 5 g, Digital Input/Output Pins: 14), shown in Figure 1a(5), connected to a PC via a cable in Figure 1a(6), two Arduino muscle v3 modules (Voltage range: ±3.5–±18 V, Gain settings: 0.01–100 kΩ, Output signal voltage: 0–+Vs) in Figure 1a(1), a 9 volt battery in Figure 1a(3) and a resistive divider in Figure 1a(4). The signal is obtained using the six electrodes (Figure 1a(2)), which are positioned on both masseter muscles of the subject (Figure 1b(1,2)). Specifically, one is positioned on the central part of the right muscle (red), one at the end of the right muscle (green) and one (yellow) on a bone not involved in movement (right cheekbone). The other three are positioned on the left side in the same way. It is possible to see the circuit diagram and the correct placement of the electrodes in Figure 1a,b, respectively.

As demonstrated in [25], the data collected using EMG technology, from which the masticatory features are extracted, are repeatable and reproducible. Regarding the noise of the signal, the muscle v3 modules are used because they contain a circuit with diodes to rectify the signal, an active low-pass filter to eliminate noise and another active amplifier to obtain an accurate signal (Muscle_Sensor_v3_users_manual, https://www.pololu.com/file/0J745/Muscle_Sensor_v3_users_manual.pdf, accessed on 6 June 2023).

### 2.2. Sample Preparation and Characterization

In this study, Conad bread is used as a food sample and is pre-cut into cubes of $1\;cm^{3}$. The hardness (Young’s modulus) of the bread is equal to 0.87 N/m and is calculated using the “univert mechanical tester” tool. Table 1 shows the grams of macronutrients in this food sample.

While seated comfortably, the subjects are asked to eat the sample. They are asked not to speak and not to move during the recording, and, finally, they are asked to indicate with a gesture when they have completed chewing. In addition, they are asked to freely consume the food following their typical chewing style. All the subjects provide voluntary informed consent to participate in the study and are informed about the ingredients present in the food administered to avoid allergic reactions.

First, the electrodes are placed as described in Section 2.1. Then, the chewing signals in tension $v_{dx}\;(t)$ and $v_{sx}\;(t)$ are acquired, which represent the electromyographic signal of the right and left masseter, respectively. Finally, these data are processed through Python software. In the algorithm, raw signals from the right $v_{dx}\;(t)$ and left $v_{sx}\;(t)$ masseter are analyzed separately and then averaged with the masticatory features defined later.

Before analysis, the signals $v_{dx}\;(t)$ and $v_{sx}\;(t)$ are rectified, amplified and filtered by the circuit modules. To eliminate any bias, the mean value of the first five samples acquired while the subject is not chewing is respectively subtracted from each signal.

**Figure 2:**
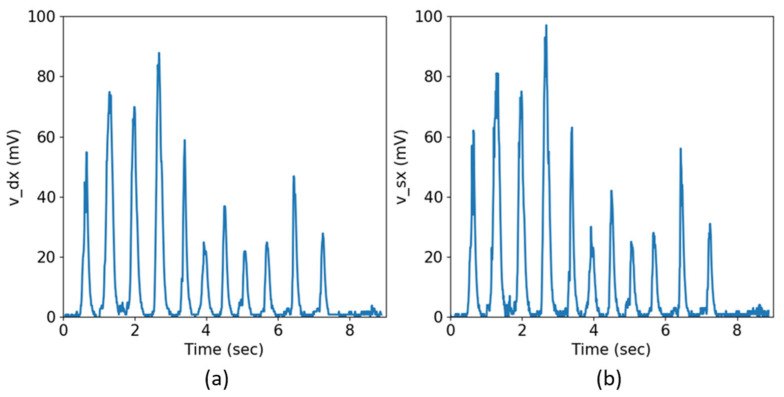
Chewing registration of the signals $v_{dx}(t)$ and $v_{sx}(t)$. The signals shown in the figures have been rectified, amplified and filtered by the circuit modules, and any bias has been eliminated via software. (**a**) Right masseter activity $v_{dx}(t)$. (**b**) Left masseter activity $v_{sx}\left(t\right)$.

Figure 2 shows an example of signal recordings $v_{dx}(t)$ and $v_{sx}(t)$ of a subject under test while eating the bread sample (respectively (a) and (b)). Each peak represents the masseter contraction.

The sampling time of the signal acquired is Δt=10  milliseconds. For $v_{dx}\;(t),$ we define the time interval of a ${∆t}_{chew\_dx}=t_{e}-t_{s}\;$ where $t_{e}$ is the ending time and $t_{s}\;$ is the starting time. We define the threshold $\sigma_{dx}^{*}\;$, which is the standard deviation of the first five samples of $v_{dx}\;(t)$ before the subject starts to chew. So, the time $t_{s}$ is determined as the value in which two consecutive $v_{dx}\left(t_{s}\right)$ and $v_{dx}\left(t_{s}+\Delta t\;\right)\;$ exceed $\;\sigma_{dx}^{*}$. The time $t_{e}$ is determined as the value by which $v_{dx}\left(t_{e}\right)>\sigma_{dx}^{*}$ and $v_{dx}\left(t_{e}+\Delta t\right)<\sigma_{dx}^{*}$. This condition is verified by performing a cycle for every t of the signal $v_{dx}\;\left(t\right).$ The same is performed for $\;v_{sx}\;\left(t\right)$.

From each time series, the chewing behavior is described in terms of five features: ‘Chewing time’, ‘Number of chews’, ‘Cycle time’, ‘Work’ and ‘Work rate’ [25]:

1. Number of chews ($n_{chew}$ adimensional): the number of chews made by the subject (). The number of detected chewing cycles ${∆t}_{chew\_dx}$ is called $n_{chew\_dx}$. The whole process is repeated to calculate $n_{chew\_sx}$. The average of $n_{chew\_dx}$ and $n_{chew\_sx},\;$ gives an estimate of the number of chews $n_{chew}$:
  (1) $$n_{chew}=\frac{\left(n_{chew\_dx}+n_{chew\_sx}\right)}{2}$$
2. Cycle Time ($t_{cyc}$, second): the time spent on a single bite in seconds. $t_{cyc\_dx}\;$ is calculated as the ratio of the sum of all the time intervals of the chews ${∆t}_{chew\_dx}\;$ and the number of chews $n_{chew\_dx}$. The whole process is repeated to calculate $t_{cyc\_sx}$. The average of $t_{cyc\_dx}$ and $t_{cyc\_sx}$, gives an estimate of the cycle time $t_{cyc}$. This parameter is a good estimate of the chewing rate (in seconds). In the following, the full formula used to calculate $t_{cyc}\;$ is reported.
  (2) $$t_{cyc}=\frac{\left(t_{cyc\_dx}+t_{cyc\_sx}\right)}{2}=\frac{\left(\frac{\sum {∆t}_{chew\_dx}}{n_{chew\_dx}}\right)+\left(\frac{\sum {∆t}_{chew\_sx}}{n_{chew\_sx}}\right)}{2}$$
3. Chewing Time ($t_{chew}$, second): the effective time in which the subject has chewed in seconds (), as expressed by the product between the number of chews and the cycle time, calculated according to the following equation:
  (3) $$t_{chew}=\frac{\left(t_{chew\_dx}+t_{chew\_sx}\right)}{2}=\frac{\left(n_{chew\_dx}\cdot t_{cyc\_dx}\right)+\left(n_{chew\_sx}\cdot t_{cyc\_sx}\right)}{2}$$
4. Work (w, volts * second): the estimated area under the masticatory signal. Right work $w_{dx}$ is the sum of the products between the mean voltage ($\bar{v_{dx}})$ and ${∆t}_{chew\_dx}.\;$ Dually, it is calculated as ${\;w}_{sx}$. The average between $w_{dx}$ and $w_{sx}\;$ is the work w.
  (4) $$w=\frac{\left(w_{dx}+w_{sx}\right)}{2}=\frac{\left(\sum \left(\bar{v_{dx}}\cdot {∆t}_{chew\_dx}\right)\right)+\left(\sum \left(\bar{v_{sx}}\cdot {∆t}_{chew\_sx}\right)\right)}{2}$$
5. Work rate (wr, volt): indicates the power exerted by the masticatory muscles (in volts), which is expressed as the ratio between the work and chewing time. This feature is calculated as the ratio between the work and chewing time:
  (5) $$wr=\frac{\left({wr}_{dx}+{wr}_{sx}\right)}{2}=\frac{\left(\left(\frac{w_{dx}}{t_{chew\_dx}}\right))+(\left(\frac{w_{sx}}{t_{chew\_sx}}\right)\right)}{2}$$
6. Asymmetry index ${\;i}_{as}$: is related to the number of chews of the masticatory, assessing whether it is balanced or not, and is calculated as follows:
  (6) $${\;i}_{as}=mean(n_{chew\_dx})-mean(n_{chew\_sx})$$

A chewing flag is associated with the index, distinguishing between:

- balanced if ${-1<i}_{as}<1$,
- slightly unbalanced to the right if ${0<i}_{as}<5\;$ or to left if ${-5<i}_{as}<0$
- unbalanced to the right $i_{as}>5\;$ or to the left if $i_{as}<-5$

### 2.3. Statistics

In this work, a case study to assess the chewing profiles of smokers and non-smokers was conducted to test the chewing pattern evaluation. To quantify the differences between the two groups through statistical analysis, the *t*-test and the Mann–Whitney test were used to clarify the relationship between the chewing behaviors and characteristics of the subjects involved in the work. Also, the FDR correction was performed to minimize the risk of obtaining false positive results when many hypothesis tests were performed simultaneously. The significance level was set at *p* = 0.05. The statistical power of the study was also determined using the “smp.NormalIndPower” function from the statsmodels library (stats-models.stats.power.NormalIndPower.solve_power). This function takes three inputs: the effect size, which is calculated as twice the U test statistic divided by the product of the sample size, as the features follow a normal distribution; the number of samples (22); and the alpha level (0.05). The resulting statistical power is equal to 0.8 (0.7 for the number of chews and 0.9 for work), indicating a high likelihood of detecting true effects in the study. The test included 25 subjects, but 3 subjects were excluded from the analysis because they presented more than one masticatory feature equal to zero (this indicates that the subject did not correctly chew the test food but directly swallowed it). The other subjects were divided into two categories: 0 are smokers (14) and 1 are non-smokers (8).

## 3. Results

### 3.1. Study Population

**Figure 3:**
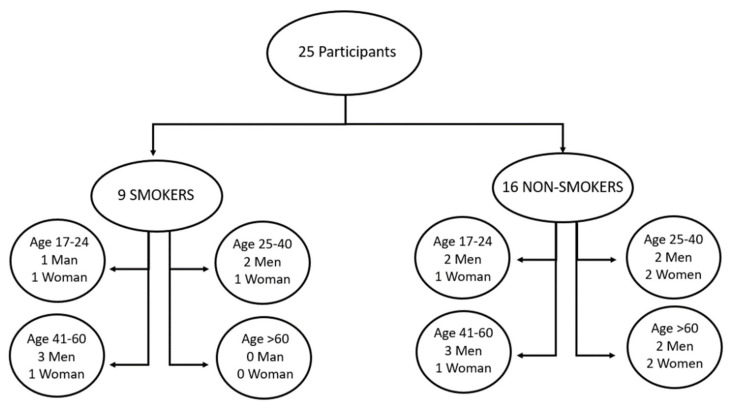
Characteristics of the group of people undergoing the test. The 25 subjects were divided into two macro-categories: smokers (9) and non-smokers (16). Each macro-category was divided into four sub-categories based on the age of the subjects (17–24, 25–40, 41–60, >60), of which the number of female (women) and male (men) subjects is indicated.

We involved 25 subjects (16 men and 8 women) with an average age of 44.04 ± 16.92 years (range 17–80 years) and an average body mass index (BMI) equal to $25.05\;\pm \;2.83\;kg/m^{2}$. The subjects were divided into two groups: smokers (0) and non-smokers (1), as shown in Figure 3. Each category was divided into four sub-categories based on age (17–24; 25–40, 41–60, >60), and the number of female (women) and male (men) subjects in the various sub-categories is indicated.

### 3.2. Chewing Profiles of Smokers and Non-Smokers

**Figure 4:**
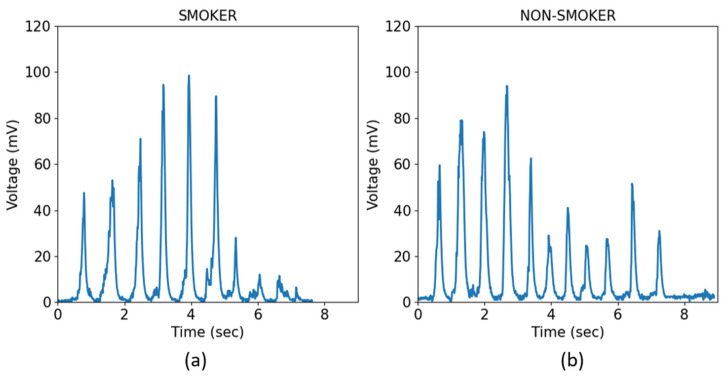
Examples of the chewing profiles of a smoker and a non-smoker. (**a**) Chewing profile for the sample of bread of a smoker; (**b**) Chewing profile for the sample of bread of a non-smoker. Chewing time and number of bites appear to be greater in the pattern of (**b**).

Figure 4 shows two representative chewing profiles of a smoker and a non-smoker who chewed the tested food (bread). It can be seen from these two profiles that the $n_{chew}\;$ and $t_{chew}$ of the non-smoker are higher than those of the smoker (greater number of peaks and time of chewing).

A complete statistical analysis of the chewing characteristics detected by our device has produced the results shown in Table 2.

The non-smokers bite more often ($n_{chew}$ = 6.31 for smokers and 9.8 for non-smokers, *p* = 0.02), the work is higher (w = 0.06 V*sec for smokers and 0.11 V *sec for non-smokers, *p* < 0.01) and the chewing time is higher ($t_{chew}$ = 3.15 s for smokers and 4.39 s for non-smokers, *p* < 0.1) than the smokers. However, these results indicate that what is significant between the two groups is not the chewing time but rather the number of chews and the mechanical work exerted to break up the food.

**Figure 5:**
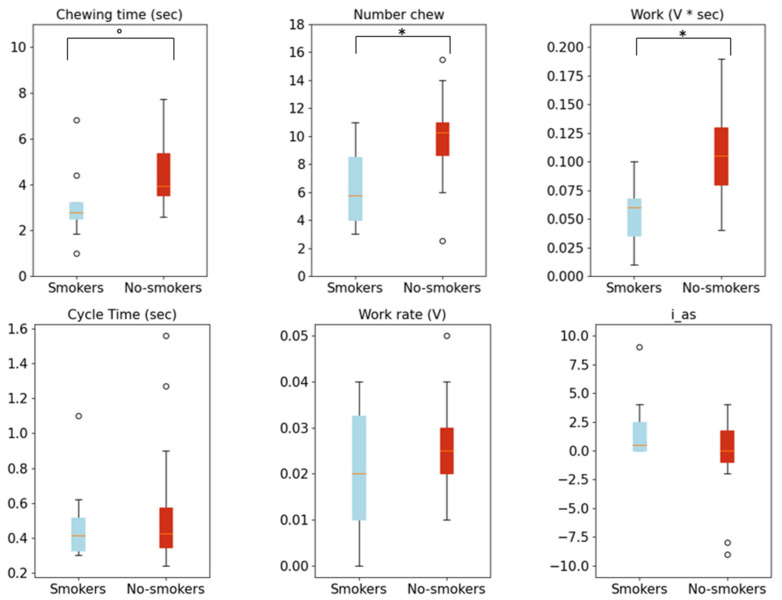
Boxplots of the chewing features of smokers (in blue) and non-smokers (in red): in the first row of the figure, there are the boxplots of chewing time, number of chews and work features; in the second row, there are boxplots of cycle time, work rate and asymmetry index. The asterisks represent a significance level ≤ 0.05. The dot represents a significance level ≤ 0.1.

Also, the statistical differences reported in Table 2 are represented as boxplots in Figure 5, where the smokers are represented in blue and the non-smokers are represented in red.

### 3.3. Graphical Clustering of the Chewing Profiles of Smokers and Non-Smokers

**Figure 6:**
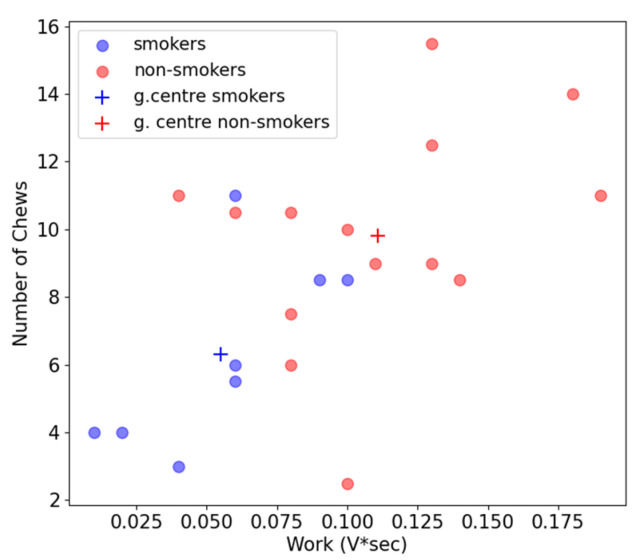
Representation of the data distribution in 2D space realized with ${\;n}_{chew}$ and *w*. The crosses in blue and red represent the geometric center of the distribution of smokers and non-smokers, respectively.

Furthermore, the device allows one to perform a clusterization in this space of the masticatory features. In Figure 6, the smokers are shown in blue, and the non-smokers are shown in red. A visual inspection of the graphs shows that the non-smokers are placed on the right side of the space, having a higher number of chews and higher work values than the smokers. Furthermore, it is possible to observe the presence of two crosses, which represent the geometric centers of the two distributions: the smokers (blue) and non-smokers (red). Specifically, the geometric center of the smokers has a number of chews of 5.71 and work of 0.05 V*sec, while the geometric center of the non-smokers has a number of chews of 8.97 and work of 0.10 V*sec.

## 4. Discussion

We have presented an innovative device, “Chewing”, which allows one to carry out a chewing analysis in a non-invasive and objective way. Compared to the other devices used for this scope, “Chewing” exploits EMG technology [18]. Once the data has been obtained, it is able to analyze them automatically, extracting the features of interest and producing a complete masticatory profile based exclusively on the performance itself. In the previous works described [22,24], the only masticatory variable considered was the speed, and, furthermore, no instrument was used to measure it. These problems have been overcome using “Chewing”, mainly for two reasons: (1) different masticatory features are defined (chewing time, number of chews, masticatory work, masticatory power, time for a single bite) and (2) it uses a gold standard technology for the evaluation. Although the use of the electrodes is uncomfortable because there are several cables connecting them to the device, this problem could be overcome by using wireless data transmission technologies (such as NFC or others).

Furthermore, our device has been tested on a use case: we considered smokers and non-smokers. The results obtained from this analysis showed how the chewing pattern of non-smokers is characterized by a higher number of chews (*p* = 0.02) and work (*p* = 0.01). These results suggest that smokers have an inefficient chewing pattern. Indeed, smokers take fewer bites than non-smokers. In relation to this aspect, in [24], it was shown that the chewing pattern of non-smokers allows for the ingestion of larger and less saliva-moistened fragments, resulting in greater effort during chewing and swallowing, which may be accompanied by compensatory movements of the head and face muscles. Similarly, here, we observed that the work performed by the smokers was less than that of the non-smokers, again indicating that they worked the bolus very little before swallowing.

In addition, smoking impacts olfactory and gustatory sensory perception. It can structurally and functionally alter the ability to perceive different stimuli in a subject. Previous studies have already demonstrated that a smoker’s ability to recognize taste is lower than that of non-smokers, with the need to increase the concentration of the tested stimulus to be recognized correctly. This results in variations in an individual’s acts of chewing and digestion. Olfactory and gustatory stimulation allows the preparation of the oral and gastrointestinal motor apparatus for the reception of food. This stimulation induces an increase in salivary secretion and gastric juice, favors the correct positioning of the oropharyngeal structures for swallowing and generates nervous and muscular excitability for the passage of food into the stomach [22]. It has also been explained how this difficulty occurs due to changes in the shape, number and vascularity of the taste buds that affect sensing ability and the perception of taste by inducing an increase in the sensory recognition threshold [22]. Continuous exposure to smoke can result in structural and functional changes in the neuroepithelium, leading to a decrease in the production of sensory cells and impaired odor recognition. This reduction in olfactory abilities, combined with a decreased gustatory capacity in smokers, may explain their tendency to chew for shorter durations and take fewer bites compared to non-smokers. The diminished perception of taste due to reduced sample quantities in this case study further hampers their ability to adequately sense flavors. The major limitation of this study is the number of subjects. As a future development, the authors aim to expand the participant pool to conduct additional analyses and differentiate chewing patterns among various subject categories. Increasing the number of subjects involved will allow a more comprehensive understanding of chewing behavior in different populations. Another limitation is the small quantities of food samples and the use of only one test food in the protocol. Indeed, this analysis can be considered the first exploratory analysis of the differences in terms of chewing patterns between smokers and non-smokers to be expanded using different types of foods and larger quantities.

As mentioned above, the areas of application of the tool can be different. First, it is a device useful to realize a specific chewing pattern. This result can be useful for the user himself to know and possibly improve his chewing style but also for carrying out specific studies for research purposes. Also, it can be used as a support for the diagnosis of dental problems, for example, to ascertain the presence of temporomandibular joint pain syndrome in the patient. Furthermore, in speech therapy, it can be a useful device because it could realize a further evaluation of the functionality of oro-facial muscles. In this way, the speech therapist can obtain more complete information about the user, and this allows him to set precise objectives of the therapeutic project based on the objective starting data to be monitored during treatment. The device could also be integrated into environments analyzing metabolic digital twins, such as Personalized Metabolic Avatar (PMA) [26,27] allowing one to estimate the subject’s energy balance and predict the weight in a personalized way.

## 5. Conclusions

In conclusion, this work demonstrates that non-smokers exhibit a more extensive and efficient chewing pattern compared to smokers, as evidenced by the higher values regarding the number of chews and work. The developed device provides valuable insights into personalized chewing profiles and can potentially contribute to modifying unhealthy chewing habits. In addition, it is non-invasive, requiring only the application of electrodes on masticatory muscles. The system presented in this paper is an alternative tool to characterize the user’s chewing and make him aware of his eating habits.

## Figures and Tables

**Table 1:** Macronutrients of the food used in the test (bread).

Food	Salt (100 g)	Fats (100 g)	Carbohydrates (100 g)	Proteins (100 g)	Sugars (100 g)	Fiber (100 g)
Bread	0	6.5	58	10	4	2.5

**Table 2:** Results of the statistical analysis.

Features	Mean Smokers	Standard Dev. Smokers	Mean Non-Smokers	Standard Dev. Non-Smokers	Statistical Test	*p*-Value ^1^
Age	43.88	14.4	46.79	20.73	−0.33	0.32
Sex	0.62	0.48	0.64	0.48	0.14	0.49
BMI	25.64	3.02	24.38	2.02	1.11	0.16
$t_{chew}$	3.15	1.66	4.39	1.39	−1.78	**0.07 °**
$n_{chew}$	6.31	2.6	9.82	3.13	−2.56	0.02 *
$t_{cyc}$	0.5	0.25	0.57	0.38	53	0.34
w	0.06	0.03	0.11	0.04	−3.12	0.01 *
wr	0.02	0.01	0.03	0.01	−0.99	0.17
${\;i}_{as}$	2	2.96	−0.57	3.66	37	**0.14**

^1^*t*-test and Mann–Whitney test based on Shapiro’s test for data normality. Statistically significant differences are reported in bold. (*) stands for *p*-value < 0.05; (°) stands for *p*-value ≤0.1

## Data Availability

The data presented in this study are available upon request from the corresponding author.
